# Characterization of the light and flexible nonlead aprons as an alternative to Pb–PVC

**DOI:** 10.1002/acm2.70398

**Published:** 2025-11-27

**Authors:** Mansour Tayebi khorami, Zaker Salehi

**Affiliations:** ^1^ Department of Radiation Sciences School of Paramedical Sciences Yasuj University of Medical Sciences Yasuj Iran

**Keywords:** diagnostic radiology, flexible lead‐free aprons, lightweight aprons

## Abstract

**Background:**

Radiation shielding is crucial for protecting healthcare professionals from scatter radiation during x‐ray procedures. Conventional lead aprons, although effective, are limited by their high weight, low flexibility, and potential toxicity. Recent developments in composite materials using elements such as tungsten (W), bismuth (Bi), tin (Sn), antimony (Sb), and barium (Ba) offer promising nonlead alternatives with comparable radiation protection, while significantly improving both weight reduction and flexibility.

**Purpose:**

This study evaluates various applicable materials in lead free radiation shielding, including W, Bi, Ba, Sn, Gd, and Sb composites to determine the weight reduction in these types of aprons. By investigating the materials' compositions for radiation attenuation, the present study aims to contribute to the ongoing development of safer, lighter, and more flexible x‐ray shielding solutions.

**Methods:**

In this study, the mass and thickness of various nonlead shielding aprons were calculated for two standard protection levels included 0.35 and 0.5 mm lead equivalence across three diagnostic energy spectrums (80, 100, and 120 kVp). Python‐based coding was employed to improve the accuracy of determining lead‐equivalent thicknesses, while MCNP was utilized to evaluate the radiation attenuation and to simulate x‐ray spectra. The generated spectra were further validated against reference data provided by SpekPy, one of the most advanced models recommended by the AAPM.

**Results:**

The results showed that increasing the lead equivalence from 0.35 to 0.5 mm increased shield mass by approximately 30%–50% for all materials. Certain composites, such as W–Sn–Gd_2_O_3_–PVC and Bi_2_O_3_–Sn–Gd_2_O_3_–PVC, demonstrated a favorable mass, maintaining competitive protection with noticeably lower mass than traditional lead‐based shields. W–Sn–Gd_2_O_3_–PVC had the lowest mass in the 100 and 120 kVp spectra, and it had the lowest mass after Bi_2_O_3_–Sn–Gd_2_O_3_–PVC in the 80 kVp spectrum. On the other side, Bi_2_O_3_–BaSO_4_–PVC and W‐BaSO_4_–PVC composites were the heaviest shields. These findings are consistent with prior literature reporting that nonlead aprons can achieve better attenuation in the diagnostic range while reducing user fatigue. Our data further confirm that composite designs can be optimized to balance shielding efficacy and ergonomics. Compared to previous studies, our results reinforce the potential of multi‐element composites to achieve equivalent or superior attenuation performance per unit mass.

**Conclusion:**

In conclusion, nonlead radiation shields, particularly those based on W–Sn–Gd_2_O_3_‐PVC or Bi_2_O_3_–Sn–Gd_2_O_3_–PVC blends, can provide adequate radiation protection while offering substantial ergonomic benefits. Their reduced weight together with high flexibility may lower the risk of musculoskeletal strain in clinical staff, making them a viable alternative to traditional lead aprons in routine diagnostic practice.

## 1 INTRODUCTION

X‐ray radiation, while invaluable in numerous fields such as medical imaging, industrial testing, and security screening, poses significant risks to human health due to its ionizing nature. Prolonged exposure can lead to severe health issues, including cellular damage, radiation burns, and an increased risk of cancer.^1^ Consequently, effective shielding from x‐ray radiation is essential for protecting both patients and professionals working with x‐ray equipment. Traditional x‐ray shielding materials typically rely on lead due to its high density and atomic number, which efficiently absorbs radiation. However, the use of lead presents several challenges, including its toxicity, weight, and environmental concerns.^2^ Lead radiation protection aprons, exert significant biomechanical stress on the spine and intervertebral discs. These aprons, often weighing up to 6.8 kg, can impose a substantial load on the wearer's musculoskeletal system, particularly during prolonged use and movement.^3^ Studies indicate that a 6.8 kg lead apron can exert an estimated load of up to 207 N/cm^2^ on the intervertebral discs.^3^, ^4^ The weight of the apron directly increases the compressive forces on the vertebrae and intervertebral discs, potentially accelerating disc degeneration and contributing to disc herniation, especially in the lumbar (lower back) region.^5^ Prolonged wearing of heavy aprons leads to increased activity and fatigue in the trunk, neck, and shoulder muscles, resulting in chronic pain, discomfort, and stiffness.^6^ To compensate, individuals may adopt unnatural postures, further straining spinal structures and increasing the risk of musculoskeletal disorders^3^, ^7^ The prevalence of back and neck pain is notably higher among healthcare professionals who frequently wear lead aprons, with some studies reporting that over 50% of radiologists experience lower back pain and 60% of interventional radiologists report neck and lower back pain.^3^, ^5^, ^6^ These limitations have driven the search for alternative, lightweight, and nontoxic materials for x‐ray shielding applications. Newer, lighter‐weight lead‐free or lead–composite aprons aim to mitigate these effects by distributing weight more effectively.

In recent years, there has been a growing interest in the development of nonlead x‐ray shields materials that offer effective protection without the environmental and health risks associated with lead. These nontoxic, lightweight alternatives have sparked significant advancements in material science and engineering, with various compounds and composites emerging as viable substitutes. Innovations in polymers and composite materials have paved the way for more efficient, durable, and sustainable x‐ray shielding options. Recent studies highlight the promising potential of polymer‐based materials, particularly those enhanced with composites and nanocomposites has shown significant promise as potential substitutes for lead‐based materials.^8^ New materials such as barium, bismuth, and tungsten have been shown to improve the x‐ray attenuation of composites.^9^, ^10^, ^11^, ^12^ These materials can be fabricated into lightweight, flexible, and durable forms, making them ideal for use in a wide range of applications, from medical imaging equipment to portable x‐ray devices. Moreover, these materials are more environmentally friendly compared to traditional lead‐based shielding, as they often contain nontoxic elements and are less hazardous to handle or dispose of.^13^ This study evaluates various applicable materials in nonlead radiation shielding, including tungsten, bismuth, barium, tin, gadolinium, and antimony composites to determine the weight reduction in these types of aprons. By investigating the materials' compositions for radiation attenuation, this work aims to contribute to the ongoing development of safer, lighter and more sustainable x‐ray shielding solutions.

## 2 MATERIALS AND METHODS

### 2.1 Material selection

A comprehensive review of relevant literature was conducted to identify suitable nonlead materials for x‐ray radiation shielding aprons. Based on selection criteria including widespread use and low toxicity, the following materials were chosen: bismuth oxide (Bi_2_O_3_), barium sulfate (BaSO_4_), tungsten (W), tin (Sn), gadolinium oxide (Gd_2_O_3_), and antimony Oxide (Sb_2_O_3_). These materials are known for their high atomic numbers and effective x‐ray attenuation properties, coupled with low toxicity profiles.^9^, ^10^, ^11^, ^12^

### 2.2 Material composition and preparation

According to K edge absorption of Ba, W, Sn, Gd, and Sb, various combinations of these selected materials were modeled to optimize shielding performance (Table 1). Simulated material combined of two‐ and three‐component composites by mixing these particles with a polymer matrix, mimicking practical apron fabrication. Each composite apron was composed of 78% by weight of the selected materials and 22% by weight of polymer to ensure structural integrity and flexibility.

**TABLE 1:** The combination and density of modeled flexible radiation shields.

	Flexible radiation shields	Weight percentage									
		Abbreviated name	Pb	W	BaSO_4_	Sn	Bi_2_O_3_	Sb_2_O_3_	Gd_2_O_3_	PVC	Density (g/cm^3^)
1	Bi_2_O_3_–BaSO_4_–PVC	Bi–Ba	0	0	39	0	39	0	0	22	2.886
2	Bi_2_O_3_–Gd_2_O_3_–PVC	Bi–Gd	0	0	0	0	39	0	39	22	3.2
3	Bi_2_O_3_–Gd_2_O_3_–BaSO_4_–PVC	Bi–Gd–Ba	0	0	26	0	26	0	26	22	2.946
4	Bi_2_O_3_–Sb_2_O_3_–PVC	Bi–Sb	0	0	0	0	39	39	0	22	2.986
5	Bi_2_O_3_–Sb_2_O_3_–Gd_2_O_3_–PVC	Bi–Sb–Gd	0	0	0	0	26	26	26	22	3.015
6	Bi_2_O_3_–Sn–PVC	Bi–Sn	0	0	0	39	39	0	0	22	3.193
7	Bi_2_O_3_–Sn–Gd_2_O_3_–PVC	Bi–Sn–Gd	0	0	0	26	26	0	26	22	3.152
8	Bi_2_O_3_–W–BaSO_4_–PVC	Bi‐W–Ba	0	26	26	0	26	0	0	22	3.146
9	Bi_2_O_3_–W–Sb_2_O_3_–PVC	Bi–W—Sb	0	26	0	0	26	26	0	22	3.225
10	Bi_2_O_3_–W–Sn–PVC	Bi–W–Sn	0	26	0	26	26	0	0	22	3.382
11	Pb–PVC	Pb–PVC	78	0	0	0	0	0	0	22	3.46
12	W–BaSO_4_–PVC	W–Ba	0	39	39	0	0	0	0	22	3.059
13	W–Gd_2_O_3_–PVC	W–Gd	0	39	0	0	0	0	39	22	3.414
14	W–Gd_2_O_3_–BaSO_4_–PVC	W–Gd–Ba	0	26	26	0	0	0	26	22	3.064
15	W–Gd_2_O_3_–Sb_2_O_3_–PVC	W–Gd–Sb	0	26	0	0	0	26	26	22	3.139
16	W–Sb_2_O_3_–PVC	W–Sb	0	39	0	0	0	39	0	22	3.172
17	W–Sn–PVC	W–Sn	0	39	0	39	0	0	0	22	3.406
18	W–Sn–Gd_2_O_3_–PVC	W–Sn–Gd	0	26	0	26	0	0	26	22	3.288

### 2.3 Monte Carlo simulation and validation

**FIGURE 1:**
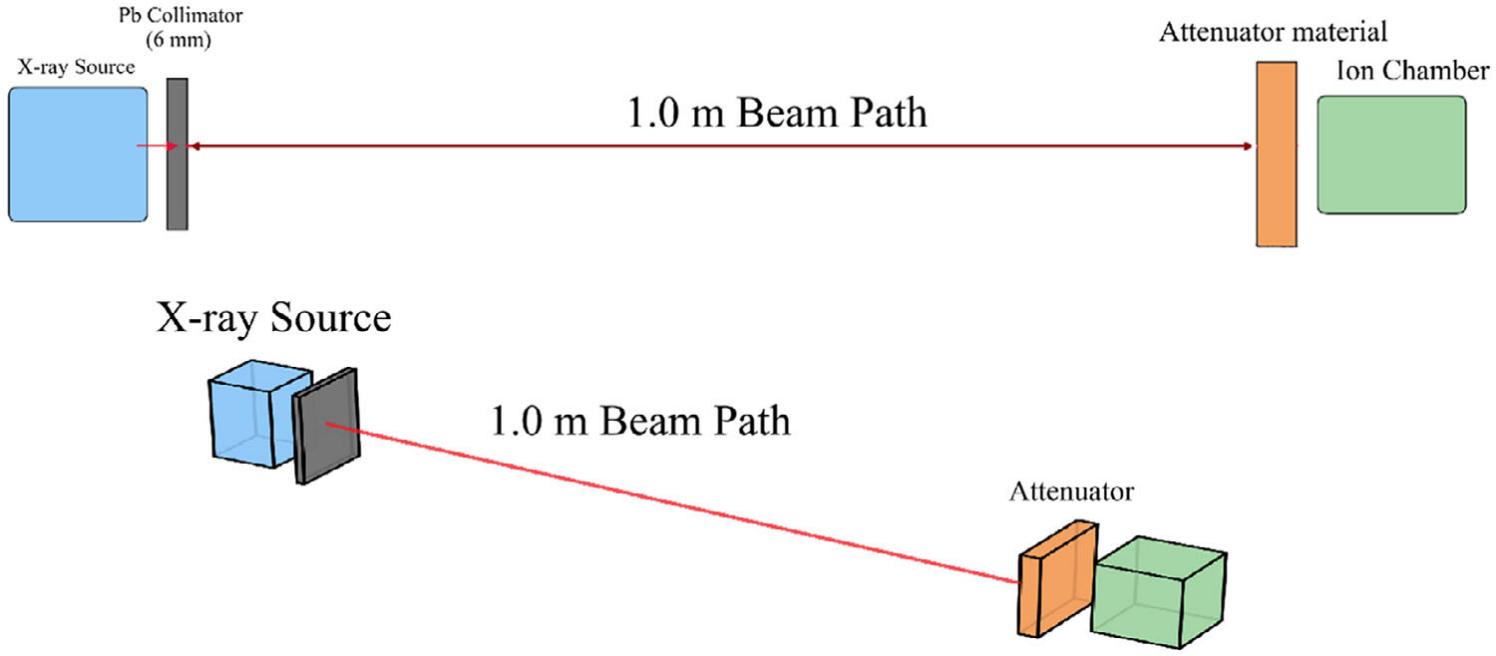
MCNP broad beam geometry set up for measurement of the transmitted radiation from several protective materials.

The shielding efficiencies of the different composite materials were calculated using Monte Carlo N‐Particle (MCNPX Version 2.6.0). For each shield configuration (nonlead composite and lead), the transmitted photon flux and energy deposition was recorded as a function of shield thicknesses. Simulations were performed for polyenergetic spectra representative of 80, 100, and 120 kVp x‐ray sources. The spectrum derived from MCNP modelling for three energies were directly compared with SpekPy V2.0, the most advanced models recommended by AAPM, for validation of the results.^14^ Attenuation curves were generated by plotting the transmitted photon flux against the shield thicknesses. The geometrical setup included a source, a layer of composite shielding material, and a detector. According to Figure 1 a fixed 100‐cm source‐to‐shield distance was maintained throughout the simulation. Photon flux and energy deposition tallies were recorded on the detector. Simulation runs comprised a sufficient number of particle histories (9 × 10⁸ histories) to achieve statistical uncertainties below 1.0% for the recorded transmission values. Standard MCNPX cross‐section libraries (MCPLIB04, EL03) were utilized, ensuring that the energy‐dependent photon interactions (photoelectric effect, Compton scattering, coherent scattering) were accurately modeled.

### 2.4 Equivalent lead thickness and mass calculation

**FIGURE 2:**
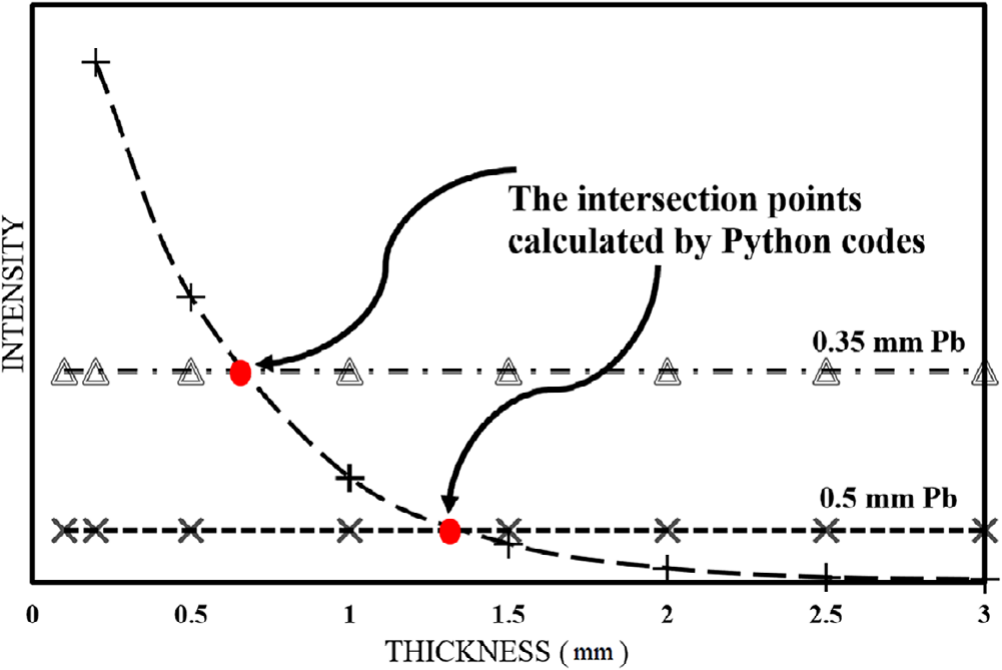
The method for calculating the lead‐equivalent thickness corresponding to 0.5 mm and 0.35 mm pure lead.

To quantitatively compare the effectiveness of the nonlead shields with that of lead, the concept of “equivalent lead thickness” was employed. After preparing the files for all three energies, the intensity of the beam passing through the 0.5 and 0.35 mm pure lead shield was extracted for all three energies. Given that each nonlead shield contains eight points (the intensity obtained after the shield with thicknesses of 0.1, 0.2, 0.5, 1, 1.5, 2, 2.5, and 3 mm), The attenuation curve was constructed by plotting the intensity of the transmitted radiation beam, as determined by MCNP simulations, against the corresponding thicknesses of the shielding materials. For the nonlead shields, eight data points were obtained and connected using exponential decay fitting to form a continuous attenuation curve, while the lead shield intensities were plotted to establish their respective curves. The equivalent lead thickness was determined by calculating the intersection points where the nonlead attenuation curve matches the intensity of the 0.5‐ and 0.35‐mm lead curves, using Python codes to numerically solve for these intersections (Figure 2). The mass per unit area is a critical parameter in evaluating the practical viability of a shielding material. A lower mass per unit area for a given attenuation level is advantageous in reducing the overall weight of protective equipment, making it more ergonomic for use in clinical settings. For a given nonlead shield configuration, the mass per unit area (kg/m^2^) was calculated based on the composite density and equivalent lead thickness. In other words, to compare the shields, the mass of one square meter of each shield was calculated based on the thickness and density.

## 3 RESULTS

**FIGURE 3:**
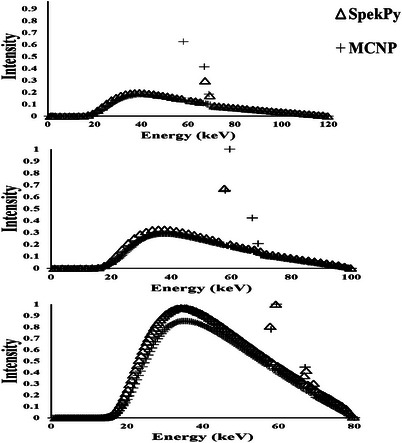
(a) comparison of x‐ray spectrum of 120 kvp tube potential from MCNP simulation and spekpy used to verify the Monte Carlo model of current study. (b) Comparison of x‐ray spectrum of 100 kVp tube potential from mcnp simulation and spekpy used to verify the Monte Carlo model of current study. (c) comparison of x ‐ray spectrum of 80 kVp tube potential from MCNP simulation and spekpy used to verify the Monte Carlo model of current study.

**FIGURE 4:**
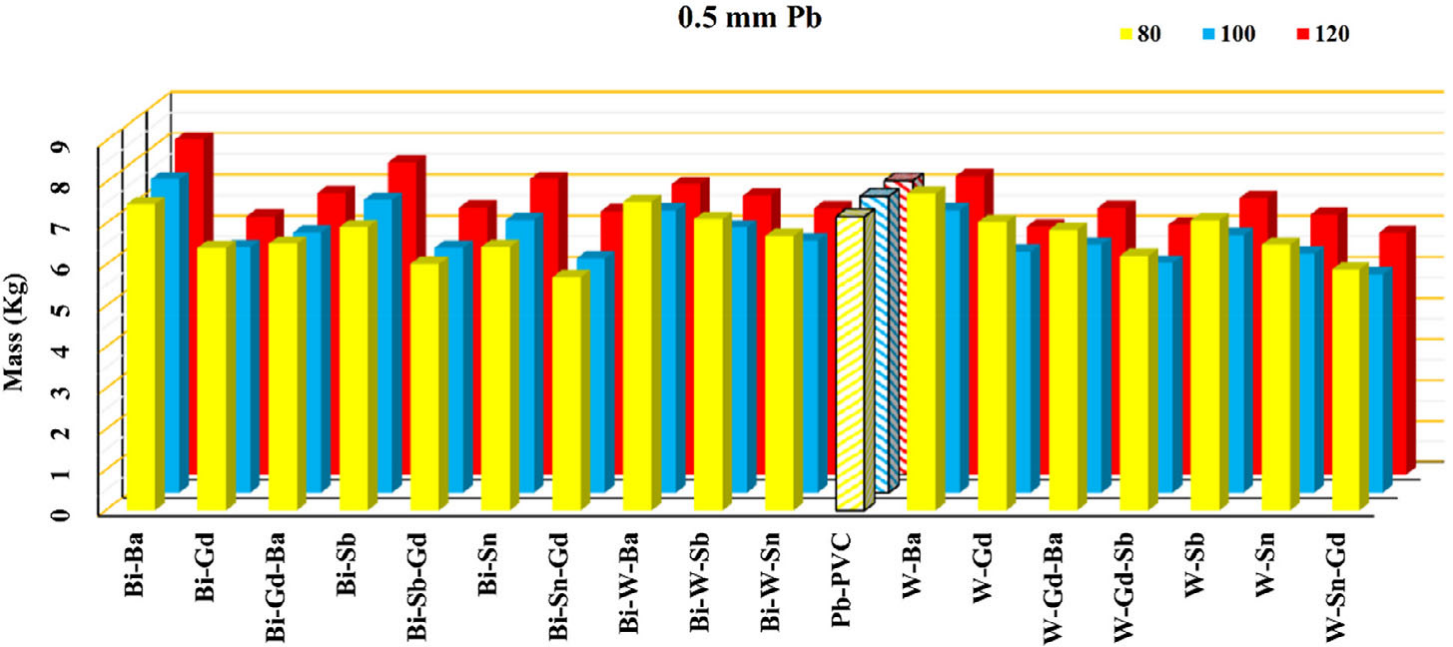
Visual comparison of the calculated mass (kg/m^2^) of 0.5 mm equivalent lead for the lead and nonlead flexible shields. All materials combined according to Table 1 and labeled with an abbreviated name.

**FIGURE 5:**
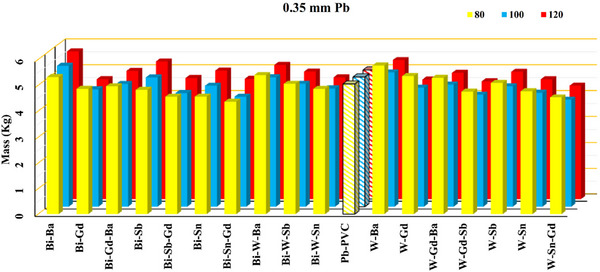
Visual comparison of the calculated mass (kg/m^2^) of 0.35 mm equivalent lead for the lead and non‐lead flexible shields. all materials combined according to Table 1 and labeled with an abbreviated name.

**FIGURE 6:**
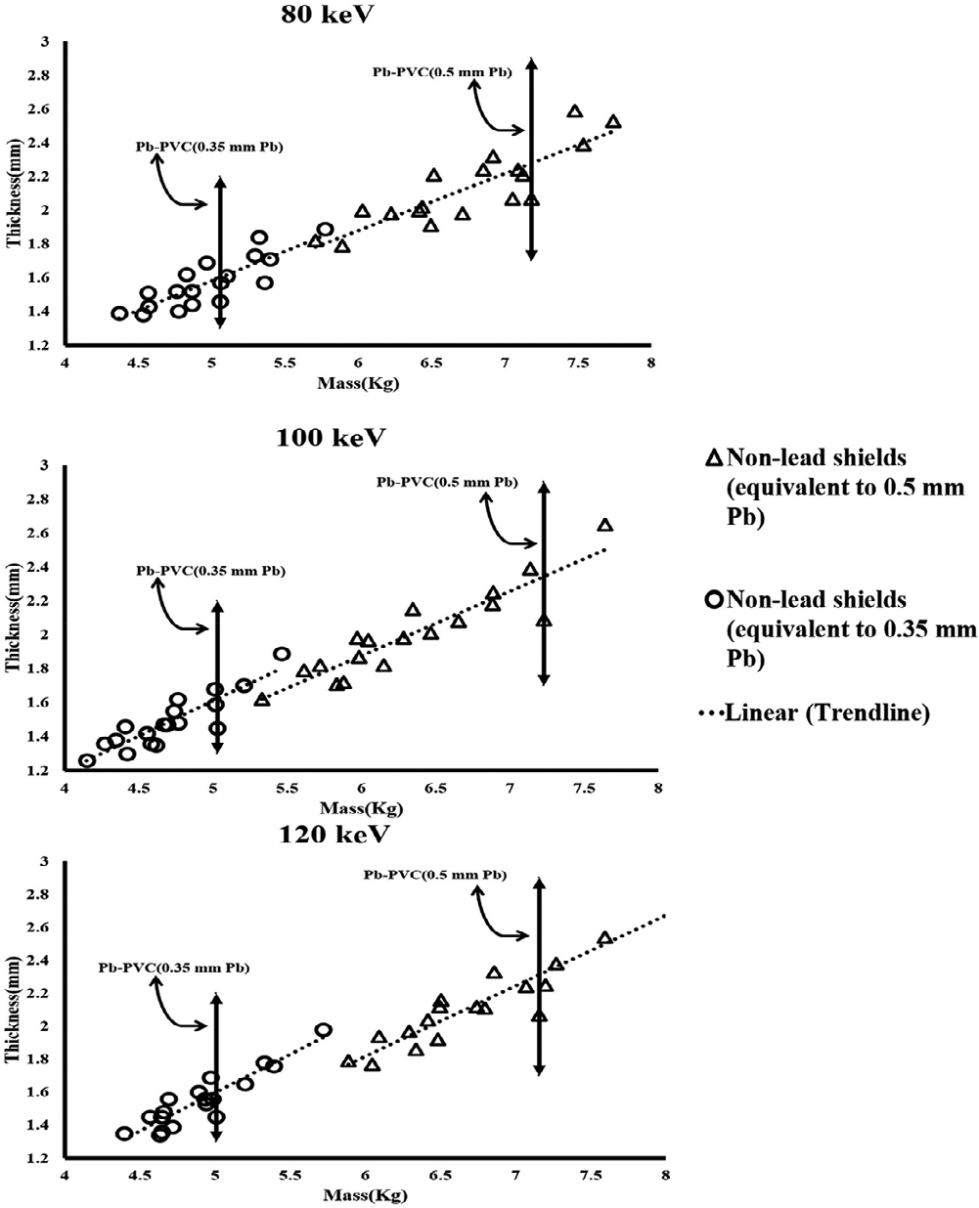
Mass versus thickness of lead and lead‐free radiation protection shields, the scatter plot compares the mass and thickness of various lead‐free radiation protection shields with two standard lead aprons (0.35 mm Pb and 0.5 mm Pb equivalence).

The Figures 3a–c illustrate the comparison between an x‐ray spectrum generated using MCNP simulation method and that obtained through an experimental approach (SpekPy). The detailed compositions of all lead and lead‐free shields are listed in Table 1. The calculated masses of 0.5‐ and 0.35‐mm Pb‐equivalent shields (both lead and non‐lead) at 80, 100, and 120 kVp are shown in Figures 4 and 5, while the corresponding calculated thicknesses are presented in Table 2. The correlation between mass and thickness for lead‐free and lead‐based aprons at 80, 100, and 120 kVp is summarized in Figure 6.

**TABLE 2:** Equivalent thicknesses (mm) of 0.5‐ and 0.35‐mm lead calculated for the lead‐free radiation shields.

	80 kVp		100 kVp		120 kVp	
	0.5 mm	0.35 mm	0.5 mm	0.35 mm	0.5 mm	0.35 mm
Bi–Ba	2.59	1.84	2.65	1.89	2.83	1.98
Bi–Gd	2.00	1.52	1.87	1.42	1.97	1.45
Bi–Gd–Ba	2.21	1.69	2.15	1.62	2.33	1.69
Bi–Sb	2.32	1.62	2.39	1.68	2.54	1.78
Bi–Sb–Gd	2.00	1.51	1.98	1.46	2.16	1.56
Bi–Sn	2.02	1.43	2.08	1.47	2.25	1.56
Bi–Sn–Gd	1.82	1.39	1.82	1.36	2.04	1.48
Bi–W–Ba	2.39	1.71	2.18	1.59	2.24	1.65
Bi–W–Sb	2.21	1.57	2.01	1.48	2.11	1.53
Bi–W–Sn	1.98	1.44	1.82	1.36	1.92	1.39
Pb–PVC	2.07	1.46	2.09	1.45	2.07	1.45
W–Ba	2.53	1.89	2.25	1.70	2.38	1.76
W–Gd	2.07	1.57	1.72	1.35	1.77	1.34
W–Gd–Ba	2.24	1.73	1.97	1.55	2.12	1.60
W–Gd–Sb	1.98	1.52	1.79	1.38	1.94	1.45
W–Sb	2.24	1.61	1.98	1.47	2.12	1.56
W–Sn	1.91	1.40	1.71	1.30	1.86	1.36
W–Sn–Gd	1.79	1.38	1.62	1.26	1.79	1.35

## 4 DISCUSSION

The Figures 3a–c illustrate the comparison between an x‐ray spectrum generated using MCNP simulation method and that obtained through an experimental approach (SpekPy). The horizontal axis represents photon energy (in keV), while the vertical axis shows normalized intensity. A close examination of the graph reveals a good level of agreement between the two methods across a broad energy range. Specifically, from approximately 15–60 keV, the simulated spectrum successfully replicates both the general shape and distribution of the experimental spectrum. The characteristic peak, which is a key feature of x‐ray emission resulting from target material interactions, appears at nearly identical energy levels in both spectra. This observation indicates that the physical processes involved in x‐ray production, namely bremsstrahlung and characteristic radiation, have been accurately modeled in the simulation. Furthermore, the continuous part of the spectrum demonstrates a consistent pattern between the two methods, reinforcing the validity of the MCNP simulation. Some minor discrepancies are observed, particularly around the characteristic peak (near 60 keV) and at higher energies beyond 70 keV. These differences may arise from several factors, including simplifications in the simulation model, uncertainties in input parameters such as tube voltage, anode composition, or geometry, and potential limitations in the experimental setup, such as detector energy response and calibration accuracy. Scientifically, MCNP modelling can be considered reliable and accurate, as it reproduces the essential features of the x‐ray spectrum with high fidelity. The close alignment with the experimental data validates the theoretical assumptions and physical models employed in the simulation. While slight deviations exist, they remain within acceptable margins for most scientific and engineering applications. In conclusion, the numerical method presented here demonstrates strong agreement with experimental results, confirming its suitability for use in scenarios where experimental measurements may be impractical or resource‐intensive. This makes it a valuable tool for spectrum prediction, optimization of x‐ray sources, and radiation shielding studies.

Figures 4 and 5 provide a comparative analysis of the mass of various nonlead radiation shields equivalent to pure lead thicknesses of 0.35 and 0.5 mm, measured at x‐ray energies of 80, 100, and 120 kVp. As shown in Figures 4 and 5, at 100 and 120 kVp the lightest shield is W–Sn–Gd, followed by W–Gd–Sb. At 120 kVp the W–Gd shield ranks third, while at 100 kVp the Bi–Sn–Gd shield takes third place. At 80 kVp, the lightest shield is Bi–Sn–Gd, followed by W‐Sn‐Gd, and the Bi–Sb–Gd is the third lightest shield at this energy. Overall, the W–Sn–Gd shield can be considered a suitable shield across all three energy spectra of 80, 100, and 120 kVp. On the side of heavier shields, the Bi–Ba and W–Ba shields were among the heaviest.

It is evident that increasing the lead equivalence thickness from 0.35 to 0.5 mm results in a considerable increase in the mass of all shielding materials. This is consistent with the direct proportionality between material thickness and mass. At 0.35 mm Pb equivalence, the mass of shields generally ranges between approximately 4–6 kg, depending on the specific material composition and energy level. The data reveal that some materials maintain relatively stable masses across varying energies, while others show more pronounced increases as the energy increases from 80 to 120 kVp. This suggests differences in material density and composition that influence the overall weight when compensating for radiation attenuation at higher energies. At 0.5 mm Pb equivalence, the masses notably increase to a range of roughly 5–8 kg, reflecting the additional material thickness required to achieve the higher attenuation standard. The upward trend in mass with increasing energy persists, indicating that shielding materials must be denser or thicker to provide effective protection at higher energy levels. Comparing specific materials reveals that certain non‐lead shields achieve similar or slightly lower masses than their lead counterparts for the same Pb equivalence, potentially offering ergonomic advantages such as reduced wearer fatigue. Pb‐PVC appear consistently near the mid‐range in mass across thicknesses and energies, suggesting balanced shielding properties and weight. Overall, these findings highlight the trade‐offs between shielding efficacy and material weight. While thicker shields provide better protection, their increased mass can impact usability. Therefore, selecting an optimal shield requires balancing protection requirements with ergonomic considerations, especially for occupational settings involving prolonged shield use.

Table 2 presents the equivalent thicknesses (in mm) of the different lead‐free shields relative to 0.5 mm and 0.35 mm Pb. According to the data, the equivalent thicknesses corresponding to 0.5 mm Pb for all tested shields range between 1.62 and 2.83 mm, while for 0.35 mm Pb they range between 1.26 and 1.98 mm. The thinnest shield equivalent to 0.5 mm and 0.35 mm Pb at 80 kVp is W–Sn–Gd (1.79 and 1.38 mm, respectively). At 100 kVp, the thinnest shield equivalence is 1.62 mm and 1.26 mm, respectively, while at 120 kVp, the thinnest shield equivalent to 0.5 mm and 0.35 mm Pb is W–Gd (1.77 and 1.34 mm, respectively). Thinner shields provide greater flexibility, which is highly advantageous for dynamic applications requiring bendability. In contrast, thicker shields may become stiff and rigid, limiting their usability in such contexts.

Figure 6 illustrates the mass–thickness relationship of the tested lead‐free radiation shields compared with the two standard lead aprons (0.35 and 0.5 mm Pb equivalence). It presents that at all energies, a linear trend is observed between mass and thickness for the non‐lead aprons, as indicated by the dotted regression line. This correlation demonstrates that for non‐lead materials, an increase in thickness is directly associated with higher mass, although the slope is less steep compared to lead‐based PVC, suggesting different density and composition characteristics. In terms of relative positioning, several nonlead shields fall **below the Pb–PVC reference line**, indicating lower thickness and mass for the same protective equivalence. These aprons are advantageous, as they provide equivalent radiation protection with reduced weight and thickness, which directly improves flexibility and comfort for clinical users. Conversely, some non‐lead shields are positioned **above the Pb–PVC reference line**, requiring greater thickness and mass to achieve the same lead equivalence. These may be less favorable for routine use due to increased stiffness and reduced wearability. When comparing the energies, the spread between the non‐lead shields and the Pb–PVC references is slightly narrower at higher energies (120 keV) compared with lower energies (80 keV). This trend suggests that the performance of certain nonlead composites becomes more comparable to lead at higher photon energies. Another important observation is that thinner and lighter shields (those positioned closer to the lower left of the plots) generally correspond to improved flexibility and easier handling. Since bendability is a critical parameter in clinical aprons, shields located below or near the Pb–PVC references on these plots represent promising alternatives. Overall, the data confirm that while some non‐lead composites still require greater thickness and mass than lead‐based aprons, a subset of materials clearly outperforms Pb‐PVC in terms of both weight and thickness, especially at 100 and 120 keV. The linear relationship between thickness and mass further highlights the consistency of material behavior across different designs, and the observed variability indicates potential for material optimization in future non‐lead apron development.

Previous study shown that at diagnostic energy levels of 60–120 kVp, non‐lead shields provide better attenuation than lead‐based counterparts, while being less toxic and similarly lightweight.^15^ This matches our observation that some non‐lead shields offer protective performance comparable to traditional lead shields while being lighter. Clinical research further supports these findings. A study comparing nonlead composites to lead aprons (0.35 mm lead‐equivalent) in interventional radiology settings showed that non‐lead 0.35 mm lead‐equivalent protective aprons may be more suitable for interventional radiology physicians, offering both effective radiation shielding and enhanced comfort in clinical environments.^16^ Our mass data support such ergonomic advantages since lower weight directly contributes to reduced physical strain. Systematic reviews also underline the growing acceptability of lead‐free aprons. They conclude that nonlead aprons incorporating elements like bismuth, antimony, and barium sulfate offer comparable protection to traditional lead, with potential benefits in mobility and reduced wearer fatigue.^17^ Finally, some studies emphasize the importance of atomic composition. Elements with favorable K‐absorption edges like Sn, Ba, Bi, and W can deliver superior attenuation per unit mass compared to pure lead under diagnostic energy spectra.^18^ This supports the design rationale behind multi‐element composite shields that aim to maximize attenuation efficiency while minimizing mass, as shown in our tested variants. Heavier leaded aprons do not offer clinically significant increased protection over thinner lead. Due to the long‐term musculoskeletal strain on interventionalists, it is safe to consider lightweight lead protection. These findings are consistent with several recent studies investigated bismuth, tin, and tungsten composites and reported the dose reduction in same lead equivalence for example, 0.25, 0.35, and 0.5 mm Pb^19^, ^20^, ^21^, ^22^, ^23^ confirming that materials like non lead composites were effective in low‐energy medical imaging environments around 40–120 keV. Together, these studies support the conclusion that several nontoxic, lightweight materials are capable of replacing lead in diagnostic radiation protection applications, particularly those designed to meet standard lead‐equivalence levels such as 0.35 and 0.5 mm Pb.

## 5 CONCLUSION

The present study has investigated the shielding performance of a nonlead composited material design to attenuate x‐rays in the diagnostic energy range (80, 100, and 120 kVp). The nonlead composite, consisting of 78% high‐density fillers (Bi_2_O_3_, BaSO_4_, W, Sn, Gd_2_O_3_, and Sb_2_O_3_) and 22% polymer, demonstrates effective photon attenuation that is comparable to that of lead shields. Our results reinforce that certain nonlead composite shields can match traditional lead in radiation protection with reduced mass, particularly at clinically relevant energies. This offers tangible ergonomic benefits, especially for practitioners engaged in long‐duration procedures. Selecting optimal materials such as W–Sn–Gd_2_O_3_–PVC, Bi_2_O_3_–Sn–Gd_2_O_3_–PVC and W–Gd_2_O_3_–Sb_2_O_3_–PVC composites enables a balanced trade‐off between shielding efficacy and user comfort. Future work should extend to long‐term wear trials and broader energy ranges to fully optimize shield design.

## AUTHOR CONTRIBUTIONS

Mansour Tayebi khorami: conceptualization, study design, contribution to methodology development, Zaker Salehi: Data Analysis, calculations, technical revision, contribution to methodology.

## CONFLICT OF INTEREST STATEMENT

The authors declare no conflicts of interest.
